# Comparative mapping and validation of multiple disease resistance QTL for simultaneously controlling common and dwarf bunt in bread wheat

**DOI:** 10.1007/s00122-020-03708-8

**Published:** 2020-10-29

**Authors:** Almuth E. Muellner, Maria Buerstmayr, Bobur Eshonkulov, David Hole, Sebastian Michel, Julia F. Hagenguth, Bernadette Pachler, Ricarda Pernold, Hermann Buerstmayr

**Affiliations:** 1 Institute for Biotechnology in Plant Production, University of Natural Resources and Life Sciences, Konrad Lorenz Straße 20, 3430 Vienna, Tulln Austria; 2 Utah State University, 2325 Old Main Hill, Logan, UT 84322 USA; 3 Present Address: Saatzucht Donau GesmbH. & CoKG, Saatzuchtstrasse 11, 2301 Probstdorf, Austria; 4 Present Address: Samarkand Branch of Tashkent State University of Economics, Professors Street 51, 140147 Samarkand, Usbekistan; 5 Present Address: Division of Plant Breeding Methodology, University of Goettingen, Carl-Sprengel-Weg 1, 37075 Göttingen, Germany; 6 Present Address: Saatbau Linz eGen, Breeding Station Schoenering, Angerweg 19, 4073 Wilhering, Austria; 7 Present Address: Mauerbachstraße 5, 1140 Wien, Austria

## Abstract

**Key message:**

Resistance QTL on chromosomes 1AL and 7AL are effective against common and dwarf bunt, QTL on 1BS affects common bunt and QTL on 7DS affects dwarf bunt in bread wheat.

**Abstract:**

Common bunt, caused by *Tilletia caries* and *T. laevis*, and dwarf bunt, caused by *T. controversa*, negatively affect grain yield and quality of wheat and are particularly destructive in low-input and organic production systems. Two recombinant inbred line (RIL) populations derived by crossing the highly and durably resistant cultivars ‘Blizzard’ and ‘Bonneville’ to the susceptible cultivar ‘Rainer’ were evaluated for their resistance to common and dwarf bunt in artificially inoculated field and greenhouse trials over two growing seasons and genotyped with a 15 K SNP array. Bunt resistance QTL were mapped to chromosomes 1AL, 1BS, 7AL and 7DS. Common bunt resistance was regulated by the major QTL *QBt.ifa-1BS* and *QBt.ifa-1AL* together with the moderate effect QTL *QBt.ifa-7AL*. Dwarf bunt resistance was on the other hand regulated by the QTL *QBt.ifa-1AL*, *QBt.ifa-7AL* and *QBt.ifa-7DS.* Common bunt resistance QTL exhibited pronounced epistatic effects, while epistatic effects were of smaller magnitude for dwarf bunt QTL. Kompetitive Allele-Specific PCR (KASP) markers were developed from SNPs associated with bunt resistance QTL and successfully used for QTL validation in an independent set of RILs. These KASP markers have the potential to support targeted introgression of QTL into elite wheat germplasm and accelerate breeding for enhanced bunt resistance. Durable protection against both common and dwarf bunt can be achieved by combining multiple resistance genes in the same genetic background.

**Electronic supplementary material:**

The online version of this article (10.1007/s00122-020-03708-8) contains supplementary material, which is available to authorized users.

## Introduction

The first historical records of bunts in wheat date back to early Greek and Roman times, and bunted heads have likely been observed since the beginning of wheat cultivation (Chen et al. 2016; Christensen 1957; Woolman and Humphrey 1924). Bunts were amongst the most destructive fungal diseases worldwide before the introduction of chemical seed treatments in the 1950s (Aboukhaddour et al. 2020; Matanguihan et al. 2011; Russell 2005). Breeding efforts for host plant resistance and research on pathogen biology have steadily decreased with the routine use of chemical seed treatment. However, bunt diseases have continued to threaten low-input farming systems and have been re-emerging in organic farming, which restricts or prohibits chemical disease management. Given the limited options for organic certified seed treatments and insufficient resistance levels of currently grown wheat cultivars in many areas of the world, breeding for bunt resistance has regained attention and become a high priority for organic wheat breeding (Borgen and Davanlou 2000; Matanguihan et al. 2011). Dwarf bunt (DB), caused by *Tilletia controversa* J.G. Kühn is restricted to regions with extended snow cover, while common bunt (CB), caused by *T. caries* (DC.) Tul. & C. Tul [syn. *T. tritici* (Bjerk.) G.Winter] and *T. laevis* J.G. Kühn [syn. *T. foetida* (Wallr) Liro] occurs in all wheat growing regions worldwide (Goates 1996; Saari et al. 1996). DB infection results from soil-borne *T. controversa* teliospores that can survive in the soil for over 10 years without losing viability, whereas CB is primarily caused by seed-borne inoculum (Borgen and Davanlou 2000; Goates 1996; Tyler and Jensen 1958). DB teliospores germinate following a preconditioning exposure to diffuse light and several weeks of stably cool temperatures of approximately 0–8 °C, conditions that are most reliably provided by an unfrozen soil surface beneath continuous snow cover (Goates and Peterson 1999; Purdy and Kendrick 1963). Germination requirements for CB teliospores are less stringent and spores germinate at a wide range of soil temperatures with an optimum of approximately 5–10 °C (Goates 1996). After infection, CB and DB fungi grow systemically in the host plant until ovary formation, after which they convert the entire endosperm within the pericarp into a bunt ball (sorus). Bunt sori are almost entirely composed of teliospores and contain significant levels of trimethylamine, which give infected grains a strong odor of rotting fish. CB and DB can cause substantial yield losses and reduce grain quality when seed-lots are contaminated with bunt balls at levels as low as 0.05% (Gaudet and Puchalski 1989).

The development and deployment of bunt resistant cultivars is the most efficient and sustainable plant protection strategy and is thus pivotal for successful organic-certified seed production and wheat cultivation in farmers' fields. To date sixteen race-specific bunt resistance (*Bt*) genes (*Bt1* to *Bt15* and *BtP*) are known (Goates 1996, 2012) thereof, only *Bt9* (Steffan et al. 2017; Wang et al. 2019), *Bt10* (Laroche et al. 2000; Menzies et al. 2006) and *Bt12* (Muellner et al. 2020) have been genetically mapped and linked markers are available for marker assisted selection (MAS). However, new pathotypes can evolve and overcome race-specific resistance genes, and new bunt races with virulence against one or more resistance genes have already been identified (Goates 2012). Consequently, cultivars that rely on single race-specific resistance genes may become susceptible due to pathogen adaptation. Pyramiding several race-specific *Bt* genes and combining them with partially effective, race-non-specific resistance QTL confers complex, horizontal resistance that provides a more durable, long-lasting resistance.

Hence, quantitatively inherited resistance is complementary to race-specific *Bt* genes. To date, seven mapping populations and three hexaploid wheat association panels have been analyzed to dissect the genetic architecture of CB resistance, identifying a total of 24 QTL on 13 chromosomes (Bokore et al. 2019; Dumalasova et al. 2012; Fofana et al. 2008; Knox et al. 2013; Singh et al. 2016; Wang et al. 2009; Zou et al. 2017). Chromosome 1B is strongly implicated in resistance, as several independent mapping populations have detected both minor and major QTL on 1B (Dumalasova et al. 2012; Fofana et al. 2008; Galaev et al. 2018; Singh et al. 2016; Wang et al. 2009; Zou et al. 2017). A wheat panel comprising 125 synthetic hexaploid wheats (Bhatta et al. 2018) and a diversity panel of 330 Nebraska winter wheat genotypes (Mourad et al. 2018) have demonstrated wide genetic variation and quantitative inheritance of CB resistance.

In comparison, genetic studies of DB resistance have been limited, likely due to the challenging nature of DB teliospore germination and subsequent disease screening. To date, only four QTL mapping studies for DB resistance have been published. Wang et al. (2019) identified a major QTL on 6DL and one on 7AL, and the QTL on 6DL most likely corresponds to *Bt9*. Chen et al. (2016) mapped a major effect QTL to the distal end of chromosome 7DS and two minor effect QTL, one on chromosome 1A and one on 2B. Muellner et al. (2020) confirmed *Bt12* as being highly effective against CB and moderately effective against DB and placed it on 7DS, in proximity to the major DB QTL reported by Chen et al. (2016). Lastly, a diversity panel of 292 wheat accessions revealed 28 accessions that were highly resistant and largely of Turkish origin and two loci associated with DB resistance on chromosome 6D (Gordon et al. 2020).

Despite clear differences in infection biology and germination requirements, the three bunt pathogens *T. controversa*, *T. caries*, and *T. laevis* are closely related and are able to hybridize (Holton 1954; Nguyen et al. 2019). This close relationship is of particular relevance in resistance breeding because host plant resistances to CB and DB are putatively controlled by the same genes in a classic gene-for-gene host–pathogen interaction framework (Flor 1956; Goates 1996, 2012; Hoffmann and Metzger 1976). The main objectives of this study were thus to (i) identify, characterize and compare QTL for resistance to CB and DB using two RIL populations derived from crosses of bunt resistant cultivars Blizzard and Bonneville to the bunt susceptible cultivar Rainer, and (ii) develop and validate KASP markers targeting CB and DB resistance QTL in an independent set of RILs to facilitate molecular breeding for improved and durable bunt resistance by pyramiding genes/QTL.

## Material and methods

### Plant material

#### Mapping populations

The two North American cultivars Blizzard and Bonneville were crossed as female parents to the Austrian cultivar Rainer to generate the two mapping populations MP-BLI and MP-BON comprising 120 and 85 F_5:7_ RILs, respectively. ‘Blizzard’ and ‘Bonneville’ are awned hard red winter wheat cultivars, highly adapted to dryland areas, and display excellent milling and baking quality according to North American standards. Blizzard and Bonneville, released in the USA by Idaho AES in 1989 (Sunderman et al. 1991) and by the USDA-ARS in 1994 (Souza et al. 1995), respectively, are closely related (Table S1, https://wheatpedigree.net/) and have maintained a high level of resistance to both CB and DB since their registration. Rainer is an awnless winter wheat cultivar released by Saatzucht Donau GesmbH & CoKG (Austria) in 2006. Rainer possesses well-adapted agronomic traits for cultivation in Austria but is highly susceptible to CB and DB.

#### Validation population

The validation population consisted of 85 BC_1_F_5_ RILs and comprised 18, 27, and 40 BC_1_F_5_ RILs derived from crossing Rainer/Blizzard//Midas, Rainer/Bonneville//20568.1.2, and Midas/Bonneville//Rainer, respectively. ‘Midas’ is an awned and locally adapted Austrian quality wheat cultivar released by Saatzucht Donau GmbH & CoKG (Austria) in 2008. The experimental line ‘20568.1.2' was selected for its high level to Fusarium head blight resistance from the cross Capo/Sumai-3. Both, Midas and 20568.1.2 are highly susceptible to CB and DB.

#### Bunt differential lines

Fourteen bunt differential lines, each carrying one of the bunt resistance genes *Bt1*–*Bt13* and *BtP* (Goates 2012), were used to monitor the virulence spectrum of the *T. caries* and *T. controversa* inoculum that was used for artificial inoculations. In accordance to Goates (2012) the reaction of the spore mixtures was considered avirulent to a specific bunt differential line when 10% or less of the spikes were diseased, and virulent if the disease incidence exceeded 10%.

### Field experiments and disease evaluations

The mapping populations MP-BLI and MP-BON were tested for CB resistance in 2015 and 2016 in two artificially inoculated field trials (CB.f15, CB.f16) and one artificially inoculated greenhouse experiment (CB.gh16) and screened for DB resistance in two artificially inoculated field trials (DB.f15 and DB.f16). The validation population was evaluated for CB resistance in one field trial in 2018 (CB.f18). RILs were grown along parental lines, control standards, and the bunt differential lines in each experiment. All trials were laid out as randomized complete block designs with two blocks (i.e. replications). For each experiment and each population, RILs were planted at *n* = 1/rep, parental lines were planted at *n* = 2/rep, and the susceptible standard ‘Capo’ was planted at *n* = 8–10/rep. Each line of the bunt differential set was planted at *n* = 1/rep. Susceptible control standards ‘Midas’, ‘Pannonikus’ and ‘Saturnus’, and resistant control standards ‘Globus’, ‘Golden Spike’ and ‘Weston’ were planted at *n* = 1/rep in all field trials. Capo is susceptibility to both, CB and DB and was chosen as the main susceptible control standard due to its extensive use in organic winter wheat production in Austria.

CB trials were conducted at the experimental station of IFA-Tulln, Austria (48°19′05′’N 16°04′10′’E, elevation: 177 masl). Originally, *T. caries* spores were collected at three different locations in Austria, representing the CB race spectrum prevalent in eastern and western Austria, and teliospores from these isolates were mixed for seed inoculation. CB spores were harvested from previous season’s infected plants displaying a typical common bunt phenotype of a diverse set of susceptible genotypes and stored under dry conditions at room temperature. Inoculation of seeds was performed according to the protocol developed by Goates (1996), applying a final concentration of 0.75 g teliospores per 100 g seeds. Each genotype was tested in a 0.5 m^2^ plot consisting of two rows, each with a length of 1.5 m and spaced 17 cm apart, with approximately 70 plants per row. CB nurseries were established in early November by sowing 6 g of inoculated, spore-coated seeds per plot. For the greenhouse experiment, inoculated seeds were germinated in early December in seedling-trays (150 plant holes per seedling-tray, top diameter of hole: 3 cm, height: 4 cm) and subjected to vernalization for eight weeks. Upon vernalization, 10 plantlets per genotype were transplanted into 7.5 l pots filled with a standard gardening soil substrate consisting of 75% heat-sterilized recycled compost, 23% peat, and 2% silica sand and moved to the greenhouse. Pots served as experimental units and were arranged in a completely randomized design with two replicates. The temperature in the greenhouse was maintained at 22/18 °C (day/night) with a 16 h photoperiod at 15,000 lx.

DB resistance was evaluated at the Utah State University Research Farm in Logan, Utah, USA (41°45′46.46″ N 111°48′54.98″ W, elevation: 1400 masl). This location is known for having long periods of snow cover, which is essential to induce high levels of dwarf bunt disease (Chen et al. 2016). Sowing took place at the beginning of October. According to a protocol developed by Goates (1996), the disease nurseries were soil inoculated after seedling emergence prior to snow cover in early November, with a water suspension of a *T. controversa* teliospore race mix, representing the virulence spectrum of races found in the USA (Chen et al. 2016). Each genotype was tested in a 1 m single row at a seeding rate of 2 g per row. Individual rows were inoculated by spraying approximately 100 ml water spore suspension containing 1.3 g spores per 1 m plot resulting in an application of between 2.5 and 3.5 × 10^8^ teliospores for each meter row. CB and DB incidence were determined as percentage of infected spikes relative to the total number of spikes within a plot at plant maturity. A spike was considered infected when containing at least one bunted spikelet.

### Phenotypic analysis

Each population was analysed separately. Best linear unbiased estimates (BLUEs) for individual environment were calculated with a linear mixed model of the form:

1 $$P_{{{ik}}} = \mu + G_{{i}} + R_{{k}} + e_{{{ik}}}$$

where *P*_*ik*_ denotes the observed phenotypic value, *μ* the population mean, *G*_*i*_ the effect of the *i*th genotype, *R*_*k*_ the effect of the *k*th replicate and *e*_*ik*_ the residual effect. The model was extended for the across environment analysis to:

2 $$P_{{{ijk}}} = \mu + G_{{i}} + E_{{j}} + E_{{j}} \left( {R_{{k}} } \right) + G_{{i}} \times E_{{j}} + e_{{{ijk}}}$$

where *P*_*ijk*_ designates again the observed phenotypic value, μ the population mean, *G*_*i*_ the effect of the *i*th genotype, *E*_*j*_ the effect of the *j*th environment, E_j_(*R*_*k*_) the effect of the *k*th replicate within the *j*th environment, *G*_*i*_  × *E*_*j*_ the *ij*th effect of the genotype-by-environment interaction and *e*_*ijk*_ the residual effect. The genotype effect was treated as fixed to derive BLUEs and random to estimate the genetic variance, while all other effects were modelled as random in both models. Fixed and random effects of the models were tested one by one using the Wald F-test. Broad-sense heritability (*H*^*2*^) was computed as suggested by Piepho and Möhring (2007):

3 $$H^{2} = \sigma^{2}_{{G}} / \, \left( {\sigma^{2}_{{G}} + \raise.5ex\hbox{$\scriptstyle 1$}\kern-.1em/ \kern-.15em\lower.25ex\hbox{$\scriptstyle 2$} {\text{ MVD}}} \right)$$

where *σ*^2^_*G*_ designates the genetic variance and MVD is the mean variance of a difference of the BLUEs. Pearson correlation coefficients were calculated between experiments and across CB and DB environments using BLUEs. All statistical analyses were conducted in R 3.1.3 (R Core Team 2016) using the package ASReml3 for mixed model analysis (Gilmour et al. 2015).

### Molecular marker analysis

Genomic DNA was extracted using a modified CTAB method (Saghai-Maroof et al. 1984). Genotyping of RILs and parents of MP-BLI and MP-BON was performed using the Illumina Infinium 15 K wheat SNP array offered by Trait Genetics GmbH (Gatersleben, Germany, https://www.traitgenetics.de) comprising 12,907 gene-associated SNPs. In addition, all lines were genotyped with SSR markers *gwm264*, *gwm374* (Röder et al. 1998), and *barc128* (Lowe et al. 2011). These markers were previously suggested to be associated with bunt resistance (Wang et al. 2009) and screened as polymorphic between the parents in pre-tests. Marker data were quality checked prior to linkage map construction and QTL mapping, whereby RILs with more than 20% missing marker data points were removed, RILs sharing more than 95% of markers were considered as the same genotype, and markers with significant segregation distortion (*p* < 0.001) and more than 20% missing values were discarded.

### Linkage map construction

Genetic maps of populations MP-BLI and MP-BON and a consensus map across both populations (MP-CON) were calculated using the statistical package ASMap v0.4 (Taylor and Butler 2017) in the R environment (The R Core Team, 2016). The objective function was set to minimize the sum of recombination events between markers for map construction. First, robust linkage groups were constructed using a stringent threshold (*p* < 1 × 10^−8^) and assigned to particular wheat chromosomes based on the hexaploid consensus wheat map (Wang et al. 2014). Within linkage groups, markers were reordered at a less stringent threshold (*p* < 1 × 10^−6^). Genetic distances were calculated with the Kosambi mapping function. Physical bp positions of mapped SNP markers were derived from the IWGSC RefSeq v1.0 annotation hosted at https://wheat-urgi.versailles.inra.fr/Seq-Repository/Annotations (Alaux et al. 2018). Graphical representation of linkage groups and QTL positions, were drawn with MapChart 2.2. (Voorrips 2002).

### QTL analysis

QTL analyses were performed with the R package R/qtl 1.46–2 (Broman et al. 2003). Genome wide QTL searches were conducted for each population separately using the population specific linkage maps and across populations using the consensus map. Analyses were conducted for CB and DB incidence BLUEs of individual experiments and the BLUEs across DB and CB field experiments. Missing genotypic information was imputed according to the multiple imputation method of Sen and Churchill (2001). In a first step, the main effect QTL were detected by composite interval mapping (CIM) using the Haley–Knott regression method. LOD significance thresholds of the respective trait, experiment and population for type I error rates at *α* < 0.1 and *α* < 0.05 were determined by running 1000 permutations. In a second step, multiple QTL models (MQM) were fitted including all significant QTL. MQM models were explored for the presence of further QTL and QTL-by-QTL interactions using *addqtl* and *addint* functions. Finally, trait and population specific MQM models were fitted including all significant QTL and QTL interactions. The overall fit of the full model against the null model was tested by ANOVA. The effect of the individual QTL were determined by comparing the full model and the model with the respective term omitted. LOD scores, estimated additive effects and percentage of the phenotypic variance explained by each QTL and QTL interaction were obtained from the drop-one ANOVA table of the MQM analysis. Confidence intervals were determined by the 1.5-LOD drop off support interval following van Ooijen (1992). QTL identified in individual populations with overlapping intervals were considered identical.

### KASP marker development

Sequence information for KASP assays of the mapped resistance loci were derived from the publicly available data set on the Cereals DB website (www.cerealsdb.uk.net; Wilkinson et al. 2016). KASP assays for SNP markers were selected based on their genetic locations on the linkage maps of the MP-BLI and MP-BON populations and on their physical positions on the IWGSC RefSeq v1.0. KASP assays were screened for polymorphism between the resistance donor and the respective recipient parent alleles and tested for co-segregation with the corresponding SNP genotype calls from the MP-BLI and MP-BON mapping populations. The full set of winter wheat bunt differential lines and a diverse set of 57 genotypes (European and international wheat cultivars, gene bank accessions and experimental lines, Table S2) were also screened with these KASP assays to verify their applicability for MAS and gene pyramiding.

### QTL validation

Markers positioned within QTL support intervals were selected for characterization of the validation population. Differences in CB incidence among genotypes grouped by QTL combination were compared with a Tukey HSD test at *p* < 0.05.

## Results

### Races’ virulence phenotypes

The *T. caries* race mix used for CB inoculations was virulent to *Bt2* and *Bt7* in field trial conditions and exhibited a broader virulence spectrum (virulent to *Bt2*, *Bt6*, *Bt7*, *Bt8, Bt9*, *Bt13,* and *BtP*) in the greenhouse. The *T. controversa* race mix used for DB field inoculations was virulent to *Bt1, Bt2, Bt4, Bt6*, and *Bt7* (Table S3).

### Trait variations and trait correlations

**Fig. 1:**
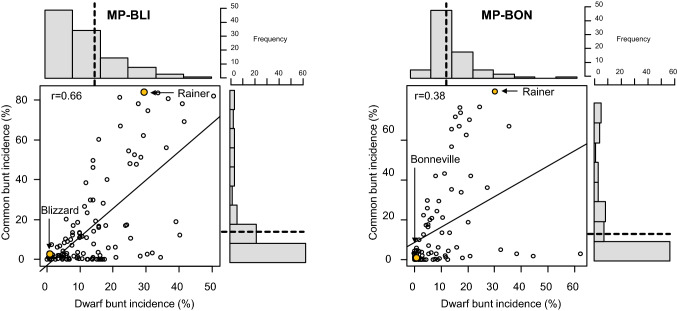
Scatterplots of BLUEs of genotypes across experiments for common bunt incidence (%) versus dwarf bunt incidence (%) with marginal histograms of the two traits in the MP-BLI (left) and MP-BON (right) mapping populations. Vertical dashed lines in histograms indicate population means

Quantitative variation was evident for CB and DB incidence in all trials, which generally followed a positively skewed continuous distribution with more than 50% of lines showing low (< 10% bunt incidence) or no infection (Fig. 1). Blizzard and Bonneville were highly resistant with BLUEs less than 1% for CB and DB incidence, whereas Rainer was highly susceptible with BLUEs of 85% and 27% for CB and DB incidence, respectively (Table 1, Table S3). Average CB incidence levels were similar across field trials and populations and ranged from 12.3 to 13%. The greenhouse trials were more diseased and had BLUEs of 26% for MP-BLI and 21.2% for MP-BON. BLUEs across populations for DB incidence varied between years, and were 9.9 and 6.3% in DB.f15 and 18.4 and 13% in DB.f16 for MP-BLI and MP-BON, respectively (Table 1). Genotype was the main source of variance for CB and DB incidence in both mapping populations (Table S4). Genetic variance was higher than genotype by environment variance, resulting in broad-sense heritabilities (*H*^*2*^) up to 0.94 for CB and 0.87 for DB incidence (Table 1). Individual experiments were highly significantly correlated in both populations (*r* = 0.95 between field experiments; *r* = 0.80–0.84 between field and greenhouse experiments). Likewise, DB experiments were highly correlated (*r* = 0.71 for MP-BLI; *r* = 0.83 for MP-BON). The correlations between BLUEs of CB and DB incidence across all experiments were *r* = 0.66 and *r* = 0.38 in the MP-BLI and MP-BON populations, respectively (Fig. 1, Table S5, S6).

**Table 1:** Parental average as well as means, minimum and maximum values, and broad sense heritability (H^2^) or repeatability (r) of the mapping populations MP-BLI and MP-BON evaluated for common and dwarf bunt incidence in 2015 and 2016

Trait	Parents			Population MP-BON					Population MP-BON				
Experiment	Blizzard	Bonneville	Rainer	Mean	Min	Max	LSD5^c^	H^2^/r	Mean	Min	Max	LSD5^c^	H^2^/r
Common bunt incidence (%)													
CB.f15	1.3	0.0	81.2	13.0	0.0	79.3	6.3	0.99^a^	12.5	0.0	83.1	7.5	0.98^a^
CB.f16	0.0	0.0	82.0	12.4	0.0	87.9	6.1	0.99^a^	12.3	0.0	80.8	7.4	0.98^a^
CB.gh16	1.9	0.0	92.2	26.0	0.0	100.0	25.2	0.92^a^	21.2	0.0	100.0	25.7	0.95^a^
Overall mean	1.1	0.0	85.0	17.2	0.0	84.2	16.8	0.94^b^	17.1	0.0	85.8	15.8	0.94^b^
Dwarf bunt incidence (%)													
DB.f15	0.3	0.0	18.5	9.9	0.0	44.7	7.7	0.93^a^	6.3	0.0	42.4	6.4	0.94^a^
DB.f16	0.9	0.5	38.8	18.4	0.0	56.0	12.2	0.89^a^	13.0	0.0	74.9	14.7	0.87^a^
Overall mean	0.6	0.3	26.9	14.2	0.0	50.4	12.4	0.83^b^	9.5	0.0	62.1	11.5	0.87^b^

^a^Repeatability

^b^Broad sense heritability

^c^Least significant differences at *α *< 0.05

### QTL analysis

#### Linkage map

After quality control, 112 RILs and 4454 markers were used to construct the MP-BLI map, resulting in 31 linkage groups (LG) with a total map length of 3228 cM. Genetic map construction for MP-BON involved 85 RILs and 5020 markers and yielded a map with 34 LG spanning 2576 cM. Seventy percent of all polymorphic markers were shared between the populations, and these 3,878 shared markers were used for calculating a consensus map across both populations. This consensus map (MP-CON) comprised 27 LGs representing 2877 cM (Online Resource Table S7).

#### QTL for bunt resistance

**Fig. 2:**
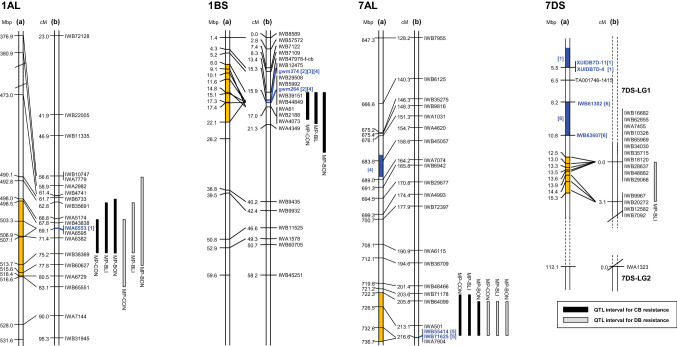
Physical and genetic maps of linkage groups containing QTL. Only relevant subsections of the chromosomes and reduced numbers of markers are shown. All linkage groups and markers with their positions are shown in Online Resource Table S7. **(a)** Mbp positions of markers are derived from IWGSC RefSeq v1.0, **(b)** genetic cM positions are obtained from MP-CON. QTL bars for CB resistance (black) and DB resistance (gray) are based on the BLUEs across CB or DB experiments of the MP-BLI, MP-BON and MP-CON populations. QTL bars span a LOD drop of 1.5 from the maximum LOD. Yellow segments within chromosome bars indicate the corresponding physical length of the QTL in the MP-CON map. Blue segments and markers highlighted in bold letters in blue color refer to positions of published QTL. Numbers in brackets designate the following refences: [1] Chen et al. (2016), [2] Wang et al. (2009), [3] Singh et al. (2016), [4] Fofana et al. (2008), [5] Wang et al. (2019), and [6] Muellner et al. (2020)

QTL analyses identified four QTL for bunt resistance that mapped to chromosomes 1A, 1B, 7A, and 7D, which were designated as *QBt.ifa-1AL*, *QBt.ifa-1BS*, *QBt.ifa-7AL,* and *QBt.ifa-7DS,* respectively. Blizzard and Bonneville are closely related and share the same allele for 73% of all mapped SNP markers. The close genetic relationship was confirmed by the low dissimilarity index of 0.12 according Bray–Curtis (Bray and Curtis 1957). Blizzard and Bonneville shared the same SNP haplotype across all QTL intervals (Table S7). All resistance improving QTL alleles descended from either Blizzard or Bonneville. QTL on 1AL and 7AL were associated both with CB and DB, whereas the QTL on 1BS only conferred resistance to CB and the QTL on 7DS was only effective against DB (Tables 2, 3, Fig. 2). The QTL on 1AL, 1BS and 7AL were detected in both populations, while the 7DS QTL was only detected in MP-BLI.

**Table 2:** Chromosomal location and estimates of QTL for common bunt (CB) incidence using multiple QTL mapping

Population			Across field experiments				CB.f15				CB.f16				CB.gh16			
Chrom	QTL interval (Mbp)		cM^a^	LOD^b^	PV%^c^	add^d^	cM^a^	LOD^b^	PV%^c^	add^d^	cM^a^	LOD^b^	PV%^c^	add^d^	cM^a^	LOD^b^	PV%^c^	add^d^
MP-BLI																		
1B	4.35	26.19	10	18.6	41.8	10.1	10	17.8	41.1	10.0	8	17.2	39.8	9.9	14	26.7	57.9	19.2
1A	492.81	515.55	68	17.0	36.9	7.7	68	15.5	34.1	7.2	72	17.2	39.8	8.8	70	19.7	36.3	14.0
7A	721.22	736.69	238	11.8	22.8	4.0	238	12.5	25.6	4.3	228	9.8	19.2	3.6				
1B:1A				4.9	8.2	5.8		3.8	6.5	5.2		5.6	10.0	6.3		8.8	12.6	10.5
1B:7A				5.0	8.3	6.0		5.7	10.1	6.1		4.9	8.6	5.4				
1A:7A				6.2	10.5	6.4		6.4	11.4	6.2		4.1	7.0	5.6				
Simultaneous fit				24.5	63.5		23.5	62.3				23.2	62.2			30.1	71.4	
MP-BON																		
1B	17.25	38.91	28	19.1	51.1	9.4	28	16.3	48.3	8.8	26	19.1	53.5	9.1	17	26.0	59.0	17.2
1A	490.09	513.89	75	12.8	28.2	7.2	75	10.3	25.2	6.5	75	12.4	28.0	6.6	74	22.1	44.1	13.7
7A	722.30	736.69	212	8.2	15.8	5.1	212	7.0	15.4	4.8	212	8.2	16.1	4.6				
1B:1A				8.0	15.3	6.1		6.3	13.8	5.8		8.1	16.0	6.0		14.6	23.0	13.9
1B:7A				5.0	8.7	4.4		4.1	8.4	4.2		5.4	9.9	4.2				
Simultaneous fit				23.4	71.9			20.1	67.3			22.9	72.3				81.3	
MP-CON																		
1B	7.99	22.07	14	33.0	41.8	9.7	14	29.8	40.4	9.3	14	31.8	42.5	9.7	18	50.4	56.9	18.4
1A	498.54	513.66	72	27.5	32.5	7.6	69	22.9	28.3	6.8	72	28.2	35.9	7.9	70	39.7	38.7	14.0
7A	722.30	736.69	210	17.7	18.4	4.0	210	17.0	19.5	4.2	210	16.2	17.6	3.7				
1B:1A				9.5	8.9	6.0		7.0	7.0	5.2		10.6	10.7	6.4		20.5	15.5	11.1
1B:7A				7.9	7.3	5.0		8.2	8.4	5.2		7.2	7.0	4.9				
1A:7A				6.7	6.1	4.3		5.8	5.8	4.2		7.1	7.0	4.6				
Simultaneous fit				43.8	64.1			39.4	60.8			41.2	62.8			59.30	75.35	

^a^Populations specific genetic position of QTL peak

^b^Log of likelihood ratio

^c^Percentage of explained phenotypic variance

^d^Positive additive effects denote trait-decreasing effect of the Blizzard or Bonneville allele

**Table 3:** Chromosomal location and estimates of QTL for dwarf bunt (DB) incidence using multiple QTL mapping

Population			Across field experiments				DB.f15				DB.f16			
Chrom	QTL interval (Mbp)		cM^a^	LOD^b^	PV%^c^	add^d^	cM^a^	LOD^b^	PV%^c^	add^d^	cM^a^	LOD^b^	PV%^c^	add^d^
MP-BLI														
1A	492.81	516.67	68	10.9	28.2	4.9	68	14.8	36.4	5.1	82	5.4	16.7	5.4
7A	722.30	736.69	250	7.0	16.5	2.9	250	8.8	19.0	2.4	250	3.3	9.8	3.1
7D	12.47	15.32	2	5.9	13.6	4.0	3	6.7	13.9	3.7	2	*2.8*	*8.3*	*3.9*
1A:7A				3.6	8.0	3.0		5.9	12.1	3.4		*0.8*	*2.2*	*2.2*
Simultaneous fit				16.9	50.1			20.3	56.5			9.6	32.5	
MP-BON														
1A	380.97	516.67	70	7.2	28.2	5.9	70	4.7	20.3	4.1	70	9.1	33.9	8.0
7A	722.30	736.69	212	3.5	12.5	3.9	212	*2.4*	*9.8*	*2.8*	212	3.1	9.8	4.3
Simultaneous fit				9.7	40.7			6.6	30.1			11.7	47.4	
MP-CON														
1A	498.54	516.67	81	16.2	28.0	5.6	74	16.7	29.2	4.9	81	13.2	23.8	6.6
7A	722.30	736.69	217	9.1	14.4	3.2	212	8.9	14.1	2.6	217	7.0	11.7	3.8
1A:7A				3.1	4.6	2.5		4.3	6.5	2.6		*1.7*	*2.7*	*2.4*
Simultaneous fit				21.4	39.5			21.0	38.7			17.9	34.4	

^a^ Populations specific genetic position of QTL peak

^b^ Log of likelihood ratio, *italic* = not significant

^c^ Percentage of explained phenotypic variance

^d^ Positive additive effects denote trait-decreasing effect of the Blizzard or Bonneville allele

*QBt.ifa-1AL* had a major effect on CB and DB across all test environments (Tables 2, 3, Fig. 2) and was positioned on the long arm of chromosome 1A close to the centromere. QTL intervals spanned a total genetic distance of 8 cM (498.5–513.7 Mbp) for CB incidence and 15.3 cM (498.5–516.6 Mbp) for DB incidence. Across both populations, phenotypic variance (PV) explained by *QBt.ifa-1AL* averaged 32.5% for CB and 28% for DB incidence and ranged from 16.7 (DB.f16, MP-BLI) to 44.1% (CB.gh16, MP-BON).

*QBt.ifa-1BS* was a constant and main component of CB incidence in both mapping populations, accounting for 40 to 59% of the observed variation, but it had no effect on DB. *QBt.ifa-1BS* was positioned on the distal end of the short arm of chromosome 1B at 8–22 Mbp in the across-population analysis (Table 2, Fig. 2). The QTL peak was closely associated with SSR markers *gwm374* and *gwm264* in both populations.

While *QBt.ifa-7AL* significantly contributed to CB resistance in all field experiments of both populations, where it explained 15.4–25.6% of the PV, it was not detected under greenhouse conditions (Table 2). The 7A QTL had a smaller effect on DB, accounting for 9.8–19% of the PV (Table 3). It was detected across all DB experiments on the MP-BLI population but was not significantly associated in the MP-BON DB.f15 trial. *QBt.ifa-7AL* mapped near the telomere of the long arm of chromosome 7A at 722–737 Mbp (Fig. 2).

The minor effect QTL *QBt.ifa-7DS* was only associated with DB in the MP-BLI population (Table 3). *QBt.ifa-7DS* mapped near the distal end of the short arm of chromosome 7D at 12.5–15.3 Mbp (Fig. 2), a region colocalizing with 16 tightly linked markers. The QTL interval was unlinked to the rest of chromosome 7D by approximately 100 Mbp due to the absence of detectable marker polymorphisms across this large interval.

**Fig. 3:**
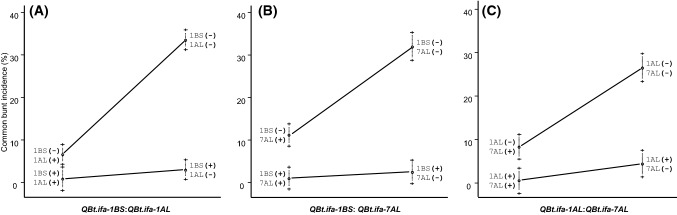
Epistatic interactions on common bunt incidence across field experiments between **(a)** *QBt.ifa-1BS*:*QBt.ifa-1AL*, **(b)** *QBt.ifa-1BS*:*QBt.ifa-7AL* and **(c)** *QBt.ifa-1AL*:*QBt.ifa-7AL*. (+) indicates the presence of the resistant allele and (−) indicates the absence of the resistant allele. Error bars of phenotypic means are plotted as ± 1 standard error

**Fig. 4:**
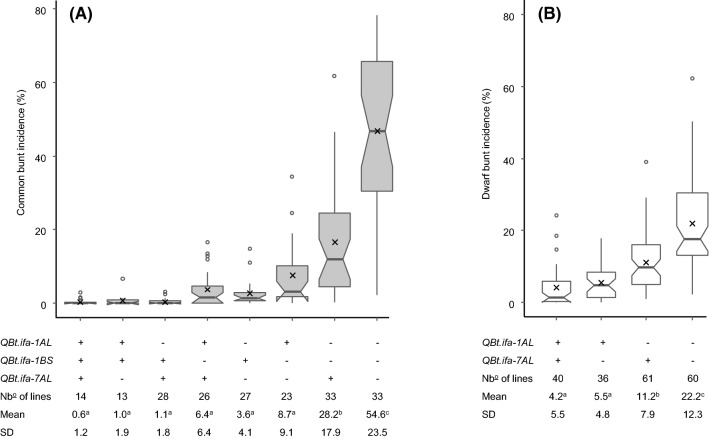
Boxplots of **(a)** common and **(b)** dwarf bunt incidence using RILS of both, MP-BLI and MP-BON populations (MP-CON), grouped by their QTL combinations. Medians are indicated by solid horizontal bold lines, means by crosses, and outliers by open circles. For each QTL combination group, QTL genotypes (+ denotes presence of resistant allele;—denotes absence of resistant allele), the number of lines, and bunt incidence means and standard deviations (SD) are provided. Groups with different letters are significantly different (*p* < 0.05) based on a Tukey HSD test. Boxplots are based on BLUEs across field trials

Epistatic interactions on CB were identified between *QBt.ifa-1BS*:*QBt.ifa-1AL *and *QBt.ifa-1BS*:*QBt.ifa-7AL *in MP-BLI, MP-BON, and MP-CON and between *QBt.ifa-1AS*:*QBt.ifa-7AL *in the MP-BLI population (Table 2, Fig. 3, Fig. 4). Furthermore, DB experiments revealed significant interactions between *QBt.ifa-1AS*:*QBt.ifa-7AL* in the MP-BLI DB.f15 experiment and in the consensus analysis across populations (Table 3).

### KASP markers and QTL validation

A set of 26 KASP markers was used for SNP haplotype screening across the *QBt.ifa-1AL, QBt.ifa-1BS, QBt.ifa-7AL,* and *QBt.ifa-7DS* QTL intervals in the resistance donors, the recipient parents, the bunt differential lines and a panel of 57 genotypes. Genotyping revealed that, depending on the KASP marker, between 12 and 83% of the tested genotypes had the same SNP call as the resistance donors Blizzard and Bonneville (Table S8). Notably, Blizzard, Bonneville and bunt differential line ‘Ridit’ (*Bt3*) shared the same SNP haplotype across the entire *QBt.ifa-1AL* interval.

**Fig. 5:**
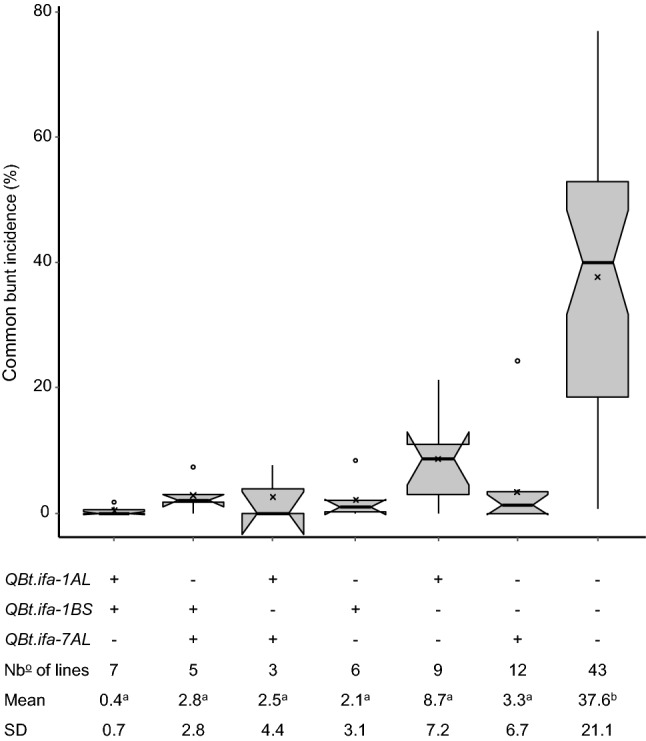
Boxplot of common bunt incidence (%) BLUEs of BC-RILs of the validation population, grouped by their QTL combinations. Medians are indicated by solid horizontal bold lines, means by crosses, and outliers by open circles. For each QTL combination group, QTL genotypes (+ denotes presence of resistant allele;—denotes absence of resistant allele), the number of lines, and bunt incidence means and standard deviations (SD) are provided. Groups with different letters are significantly different (*p* < 0.05) based on a Tukey HSD test

BC_1_-RILs of the validation population were selectively genotyped for their allele status at *QBt.ifa-1AL, QBt.ifa-1BS,* and *QBt.ifa-7AL* using four KASP markers per QTL. Lines were subdivided according to their QTL combinations, and the corresponding overall CB incidence BLUEs of grouped lines were compared. Subgroups carrying the resistance conferring allele only at single QTL or at multiple loci were all highly resistant and not significantly different from each other, with mean performances ranging from 0.4 to 8.7%. In contrast, lines without any resistance QTL showed a mean incidence of 37.7% and were significantly more diseased than all other groups (Fig. 5).

## Discussion

High levels of CB and DB incidence in the susceptible parents, check cultivars and some *Bt* differential lines confirmed that inoculation was successful and that disease pressure was high (Table 1, Table S3). CB incidence levels were higher in the greenhouse than in the field (Table 1). Similarly, Knox et al. (1998), Wang et al. (2009) and Laroche et al. (2000) reported much higher CB infection levels under controlled growth chamber than in field trials. The same CB spore mixture was used as inoculum for CB field and greenhouse trials. However, the bunt differential lines demonstrated a broader virulence spectrum in the greenhouse (Table S3), possibly due to environment dependent interactions between *Bt* resistance genes and the pathotype composition (Gaudet and Puchalski 1995). CB infection is optimal at soil temperatures of 5–10 °C (Purdy and Kendrick 1963), and sustained low temperatures during early plant development can decrease host plant resistance and subsequently increase disease incidence (Gaudet and Puchalski 1995; Griffith et al. 1955; Smith 1932; Zscheile 1956). The duration of the cold period strongly effected bunt expression (Zeischle 1956). We vernalised all seedlings at a constant temperature of 4 °C for eight weeks. Similar environmental conditions are unlikely to occur in the field. We thus hypothesize, that the specific vernalisation and greenhouse conditions probably increased disease incidence in our greenhouse experiment. Interaction between *Bt* incidence and environment has been previously reported and agree with our results (He and Hughes 2003; Knox et al. 1998; Laroche et al. 2000; Wang et al. 2009).

Sustained low temperatures, moisture and low light levels are most conducive for DB infection, and such conditions are most reliably provided by persistent snow cover (Tyler and Jensen 1958). DB infection in both populations was low in the DB.f15 trial, likely due to the shorter duration of snow cover than in the DB.f16 experiment. However, genotype was the main source of variance and heritability was high for DB and CB incidence, indicating that the observed variation among the tested lines was mainly due to genetic effects (Table 1, Table S4). The DB inoculum of US origin possessed a broader virulence spectrum than the Austrian CB inoculum (Table S3). A similar result was obtained by Huber and Buerstmayr (2006) who reported that cultivars or lines resistant to CB were not necessarily resistant to DB. It is well documented that substantial differences in the virulence spectra exist among *Tilletia* species as well as between pathogen isolates within *Tilletia* species (Hoffmann and Metzger 1976; Hoffmann 1982; Goates 2012; Goates and Bockelman 2012). Hence, a *Bt* gene/QTL conferring resistance against the currently prevailing CB or DB composite in one region may not be effective against the prevailing DB or CB composite in another region. Since almost all studies to date reported either QTL for CB or for DB resistance, efficacies of QTL for both, DB and CB resistance, should always be verified in additional experiments.

Blizzard and Bonneville have maintained excellent bunt resistance since their registration in 1989 and 1994, respectively, likely due to their possession of resistance alleles at three CB QTL and three DB QTL. The CB species *T. caries* and *T. laevis* and the DB species *T. controversa* are closely related and it has been assumed that both diseases are largely controlled by the same *Bt* genes (Goates 1996, 2012; Hoffmann and Metzger 1976; Metzger and Hoffmann 1978). But this was only partially evident in our study. *QBt.ifa-1BS* was a major source of CB resistance in both populations, explaining up to 59% of PV, whereas the same QTL had no effect on DB resistance in Utah. Differences in the virulence spectrum between the applied CB and DB inocula are the likely cause of this discrepancy (Table S3). That *QBt.ifa-1BS* specifically confers CB resistance is supported by its colocalization with a major QTL for CB resistance on chromosome 1B (Wang et al. 2009) and its lack of association with DB (Chen et al. 2016) in independent mapping populations with either Blizzard or ‘IDO444′, which is a full-sib of Blizzard, as resistance donors. Earlier studies assigned *Bt4*, *Bt5* and *Bt6* to chromosome 1B (McIntosh et al. 1998; Schmidt et al. 1969), making them putative candidate genes for *QBt.ifa-1BS*. However, KASP marker validation revealed that the bunt differential lines *Bt4, Bt5,* and *Bt6* had different haplotypes than the resistance donors Blizzard and Bonneville across the *QBt.ifa-1BS* interval, suggesting that the resistance of *QBt.ifa-1BS* differs from that of *Bt4*, *Bt5* and *Bt6* (Table S8). Several studies identified major and minor QTL for CB resistance on chromosome 1B in independent mapping populations (Dumalasova et al. 2012; Fofana et al. 2008; Galaev et al. 2018; Singh et al. 2016; Wang et al. 2009; Zou et al. 2017). Of these, QTL reported by Fofana et al. (2008), Wang et al. (2009) and Singh et al. (2016) were located near the SSR marker *gwm264* and thus coincided with the QTL region of *QBt.ifa-1BS* (Fig. 2). The resistance donors of these QTL included ‘AC Domain’ (Fofana et al. 2008), Blizzard (Wang et al. 2009) and ‘Carberry’ (Singh et al. 2016). Based on pedigree information, it remains unclear whether the CB resistance QTL on chromosome 1B from AC Domain and Carberry is identical to *QBt.ifa-1BS*. Further comparative studies are necessary to clarify this*.*

*QBt.ifa-1AL*, located on chromosome 1A at 498.5–516.6 Mbp, was a main and constant source of CB and DB resistance in both populations and in all test environments (Table 2, Fig. 2). Because *QBt.ifa-1AL* coincided with the DB resistance QTL *Q.DB.ui-1A* reported by Chen et al. (2016) and because the donor of *Q.DB.ui-1A* (IDO444, full-sib of Blizzard) is closely related to Blizzard and Bonneville (Table S1), we assume that the QTL underlying the resistance gene at *QBt.ifa-1AL* and *Q.DB.ui-1A* are identical by descent. KASP marker validation revealed that Blizzard, Bonneville and the bunt differential cultivar ‘Ridit’ (*Bt3*) had the same SNP haplotype for all eight KASP markers across the entire *QBt.ifa-1AL* interval (Table S8). Ridit expressed high but incomplete resistance (1.6–9.1% bunt incidence) in all CB and DB test environments (Table S3), similar to genotypes that were carriers of *QBt.ifa-1AL* only (Figs. 3, 4). The chromosomal location of *Bt3* is yet unknown. Given that Ridit appears in the pedigrees of Blizzard, Bonneville and IDO444, *QBt.ifa-1AL* may represent the bunt resistance gene *Bt3.*

*QBt.ifa-7AL* had a moderate effect on CB and DB incidence in all field trials but had no effect in the greenhouse experiment. This indicates that expression of specific resistance genes may depend on environmental conditions, particularly on the temperature during early plant development, and that greenhouse experiments only partially reflect the complex interplay of pathogen, host plant genotype and environment. The QTL interval of *QBt.ifa-7AL* mapped near the telomere of chromosome 7AL and coincided with the major DB resistance QTL *Q.DB.ui-7AL* derived from the DB resistant line ‘IDO835′ (Wang et al. 2019). Blizzard, Bonneville, and IDO835 share the highly bunt resistant breeding line ‘PI 476212′ (Sunderman et al. 1986) in their pedigrees (https://www.ars-grin.gov/). Thus, PI 476212 may be the resistance donor of both *QBt.ifa-7AL* and *Q.DB.ui-7AL.* This is further supported by KASP marker screening, from which Blizzard, Bonneville, and PI 476212 showed identical SNP haplotypes for all KASP markers across the 7AL QTL interval (Table S8).

*QBt.ifa-7DS* was only associated with DB in the MP-BLI population and its effect on DB incidence was relatively small (Table 3). *QBt.ifa-7DS* mapped near the distal end of the short arm of chromosome 7D, approximately 7 Mbp from *Q.DB.ui-7DS* and 2 Mbp from the bunt resistance gene *QBt.ifa-7D*|*Bt12* (Fig. 2). *Q.DB.ui-7DS* (Chen et al. 2016), *QBt.ifa-7D*|*Bt12* (Muellner et al. 2020) and *QBt.ifa-7DS* (current study) all faced the same problem of a lack of marker polymorphisms flanking the QTL, so that associated markers physically span relatively short intervals that are unlinked from the rest of chromosome 7D. These physically large monomorphic intervals, however, complicated precisely mapping of QTL positions and map comparisons. *Q.DB.ui-7DS*, *QBt.ifa-7D*|*Bt12* and *QBt.ifa-7DS* possessed the same marker haplotype at *Q.DB.ui-7DS* (*wPt-2565*) and at all markers distal to *Q.DB.ui-7DS* (Jianli Chen, University of Idaho, personal communication). Unfortunately, all markers informative for *Q.DB.ui-7DS* were monomorphic for *QBt.ifa-7D*|*Bt12* and *QBt.ifa-7DS*. There was a notable difference in effect size between *Q.DB.ui-7DS* (> 50%), and *QBt.ifa-7DS* (12%), which may be due to differences in physical positions of polymorphic markers between the two mapping populations. Markers associated with the major bunt resistance gene *Bt12* are physically positioned between *Q.DB.ui-7DS* and *QBt.ifa-7DS* (Fig. 2), but the precise location of *Bt12* is unknown as well. *Bt12* has been reported to have a major impact on CB and only a minor to moderate one on DB resistance (Muellner et al. 2020). This is in contrast to Chen et al. (2016), who reported a strong effect on DB, and our finding that *QBt.ifa-7DS* conferred resistance to DB only. Resistance of *Bt12* was derived from the *Bt* differential line ‘PI 119333′. Since PI 119333 is genetically unrelated to IDO444 and Blizzard, it remains unclear whether *Bt12* represents the same resistance allele as *QBt.ifa-7DS* and/or *Q.DB.ui-7DS.*

Epistatic interactions were substantial for CB resistance but were only weakly expressed for DB (Tables 2, 3). QTL by QTL interactions were observed in which the effect of one QTL was masked by the presence of a second major QTL, and genotypes with only one QTL appeared to be almost as resistant as genotypes that possessed two or more resistance QTL (Figs. 3, 4, 5). Epistatic interactions between bunt resistance QTL were also found by Chen et al. (2016), Knox et al. (2013) and Singh et al. (2016). Epistasis is a common phenomenon when two or more large effect resistance genes, which can replace each other, segregate in a population (also known as duplicated gene action).

The bunt resistance QTL *QBt.ifa-1BS, QBt.ifa-1AL,* and *QBt.ifa-7AL* identified in the mapping populations (Fig. 4) were successfully verified in the validation population (Fig. 5). The selected KASP markers have also been employed to track the transmission of the respective resistance QTL in an internal backcrossing program (results not shown) and are thus well suitable for marker-assisted selection (MAS). However, none of the currently available markers is diagnostic for a bunt resistance QTL. Markers are linked to the QTL and their usefulness must be tested in any given breeding program and might diminish by on-going recombination. Hence, we recommend relying on flanking markers for tracking resistance QTL to minimize the frequency of false positives during MAS and QTL introgression.

In conclusion, Bonneville and Blizzard represent modern winter wheat types, combining acceptable agronomic and quality traits with high and durable resistance to CB and DB. Bonneville is still used in dryland production, whereas Blizzard is out of production but has been used as a source of DB resistance worldwide (Jianli Chen, University of Idaho, personal communication). These cultivars share durable bunt resistance, which appears to originate from a combination of several small and large effect QTL. SNP markers linked to these QTL can be exploited for breeding a new generation of productive and bunt resistant cultivars, which should possess various combinations of resistance genes to ensure long-lasting protection against these fungal diseases.

## Electronic supplementary material

Below is the link to the electronic supplementary material.

Supplementary file1 (PDF 367 kb)

Supplementary file2 (XLSX 555 kb)
