# Supplementary material for: Comparative mapping and validation of multiple disease resistance QTL for simultaneously controlling common and dwarf bunt in bread wheat
Source: Theor Appl Genet. 2020 Oct 29;134(2):489–503. doi: 10.1007/s00122-020-03708-8 (PMC7843488; doi:10.1007/s00122-020-03708-8)
Supplement: Supplementary file 1 — Supplementary file1 (PDF 367 kb) [file 122_2020_3708_MOESM1_ESM.pdf]

Online Resource 1

Article title: Comparative mapping and validation of multiple disease resistance QTL for simultaneously controlling common and dwarf bunt in bread wheat

Journal: Theoretical and applied genetics

Authors: Almuth E. Muellner, Maria Buerstmayr, Babur Eshonkulov, David Hole, Sebastian Michel, Julia F. Hagenguth, Bernadette Pachler, Ricarda Pernold, Hermann Buerstmayr

Name, affiliation, and email of corresponding author:

Maria Buerstmayr, Department for Agrobiotechnology Tulln, BOKU-University of Natural Resources and Life Sciences-Vienna, Konrad Lorenz Str. 20, 3430 Tulln, Austria
e-mail: maria.buerstmayr@boku.ac.at

Content: Table S1 – Table S6

Table S1 Pedigree of Blizzard, Bonneville and IDO444 (http://wheatpedigree.net/)

Table S2 Wheat genotypes used for KASP marker validation

Table S3 Disease reaction of common bunt (CB) and dwarf bunt (DB) spore mixtures used for artificial inoculation

Table S4 Variance component estimates (σ^2) of genotype, environment, block, genotype by environment interaction and residual effects for CB and DB incidence in population MP-BLI and MP-BON

Table S5.1 Pearson's correlation coefficients between RIL bunt incidence BLUEs for all CB and DB environments analysed in mapping populations MP-BLI

Table S5.2 Pearson's correlation coefficients between RIL bunt incidence BLUEs for overall means across CB and DB environments analysed in mapping population MP-BLI

Table S6.1 Pearson's correlation coefficients between RIL bunt incidence BLUEs for CB and DB environments analysed in mapping population MP-BON

Table S6.2 Pearson's correlation coefficients between RIL bunt incidence BLUEs for overall means across CB and DB environments analysed in mapping population MP-BON

Table S1 Pedigree of Blizzard, Bonneville and IDO444 (http://wheatpedigree.net/)

BLIZZARD:
A68203W-E-1-3-3/A68203W-A-1-6-1 ; A68203W = Utah 216c-12-10
/Cheyenne/5/PI476212/4/Burt/3/Rio/Rex//Nebred

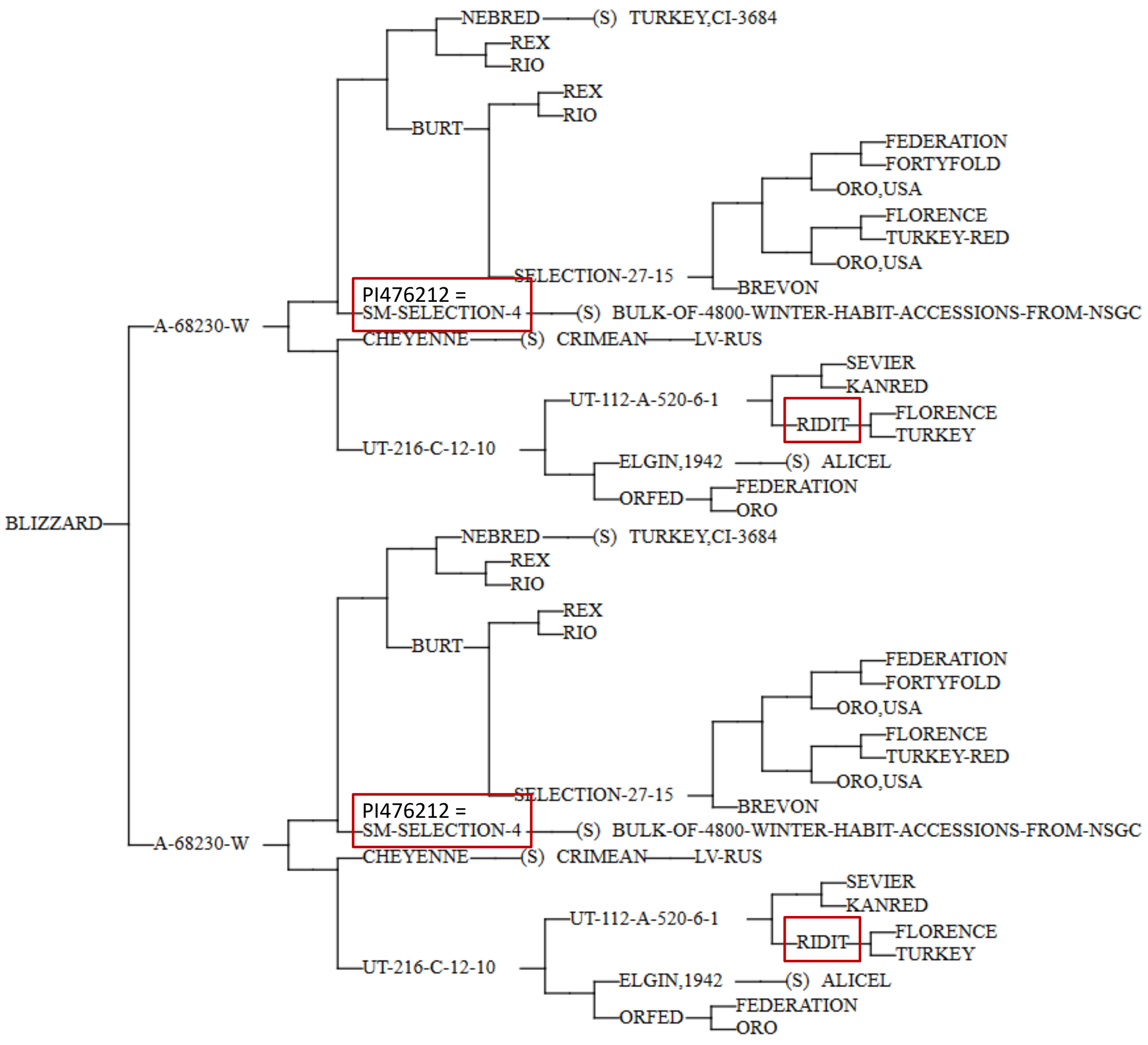

BONNEVILLE:
A774125W-16-3-1/A7470W-11-2 = Utah216c-12-10/Cheyenne/5/PI476212/4/Burt/3/Rex/Rio//Nebred/6/Kiowa/Utah222a-437-2//Dm/3/PI476212/MT6619/4/McCall/El Gaucho/3/Kiowa/Utah233-3-10/Burt

UT233-3-10: Ridit//Kanred/Sevier/3/Orfed/Elgin)/4/F1(Yogo/Wasatch
UT222-A-437-2: Yogo/Wasatch

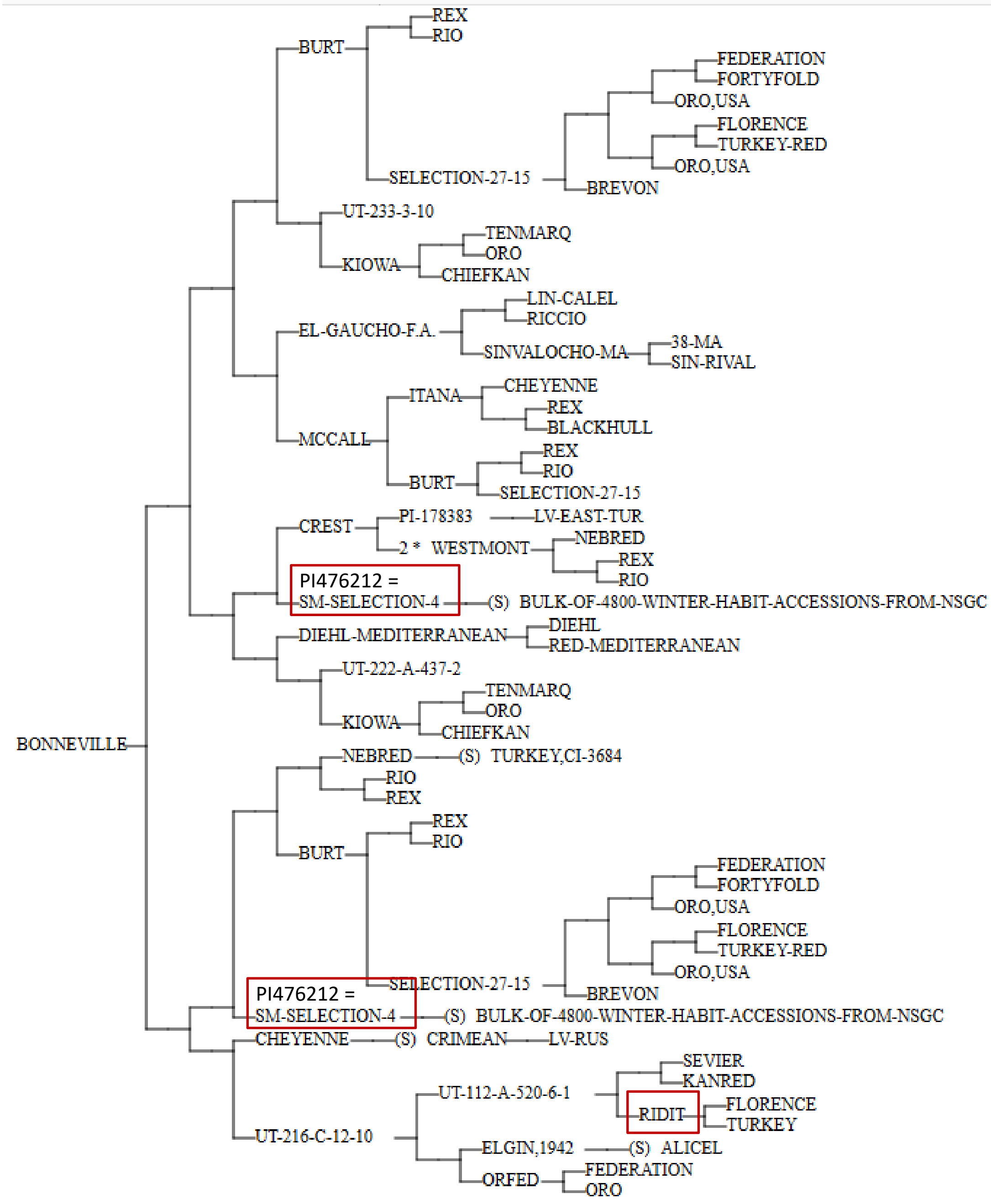

IDO444:
Utah 216c-12-10/Cheyenne/5/PI 476212/4/Burt/3/Rio/Rex//Nebred//6//Utah 216c-12-10/Cheyenne/5/PI 476212/4/Burt/3/Rio/Rex//Nebred

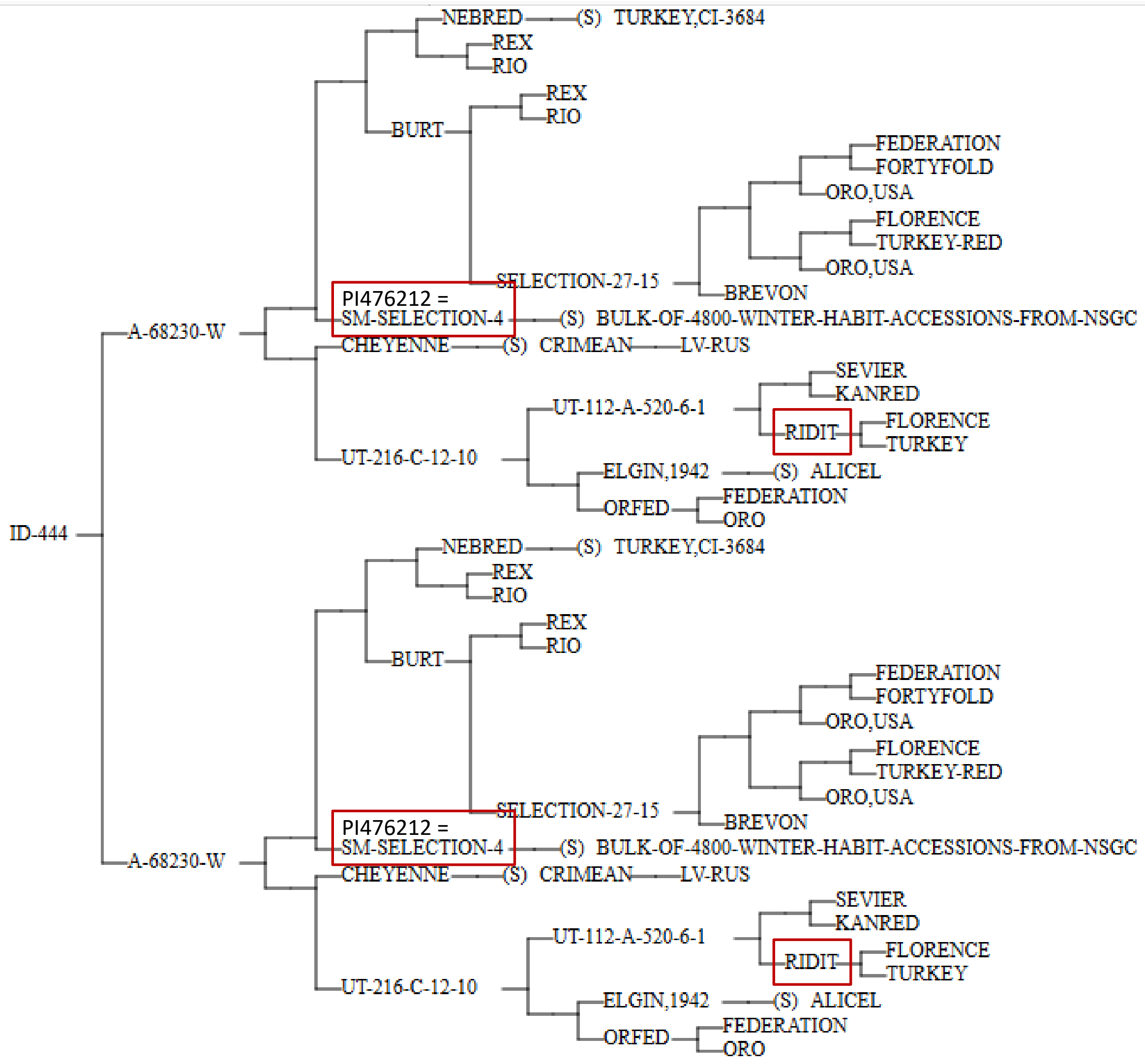

Table S2 Wheat genotypes used for KASP marker validation

Genotype Name	Category	Origin
Selection 2092	*Bt1* bunt differential line	Blair Goates, USDA, Aberdeen, Idaho, USA
Selection 1102	*Bt2* bunt differential line	Blair Goates, USDA, Aberdeen, Idaho, USA
Ridit	*Bt3* bunt differential line	Blair Goates, USDA, Aberdeen, Idaho, USA
CI 1558B	*Bt4* bunt differential line	Blair Goates, USDA, Aberdeen, Idaho, USA
Hohenheimer	*Bt5* bunt differential line	Blair Goates, USDA, Aberdeen, Idaho, USA
Rio	*Bt6* bunt differential line	Blair Goates, USDA, Aberdeen, Idaho, USA
Selection 50077	*Bt7* bunt differential line	Blair Goates, USDA, Aberdeen, Idaho, USA
M82-2161	*Bt8* bunt differential line	Blair Goates, USDA, Aberdeen, Idaho, USA
M90-387	*Bt9* bunt differential line	Blair Goates, USDA, Aberdeen, Idaho, USA
M82-2102	*Bt10* bunt differential line	Blair Goates, USDA, Aberdeen, Idaho, USA
M82-2123	*Bt11* bunt differential line	Blair Goates, USDA, Aberdeen, Idaho, USA
PI 119333	*Bt12* bunt differential line	Blair Goates, USDA, Aberdeen, Idaho, USA
Thule III	*Bt13* bunt differential line	Blair Goates, USDA, Aberdeen, Idaho, USA
PI 173437	*PtP* bunt differential line	Blair Goates, USDA, Aberdeen, Idaho, USA
Adamus	Cultivar	KWS Lochow, Germany
Adesso	Cultivar	Saatzucht Donau, Austria
Alessio	Cultivar	Saatzucht Donau, Austria
Alicantus	Cultivar	Saatzucht Donau, Austria
Amicus	Cultivar	Saatzucht Donau, Austria
Annie	Cultivar	Selgen a.s., Czech Republic
Arminius	Cultivar	Saatzucht Donau, Austria
Arnold	Cultivar	Saatzucht Donau, Austria
Aurelius	Cultivar	Saatzucht Donau, Austria
Bernstein	Cultivar	Syngenta, Germany
Bill	Cultivar	Nordsaat, Germany
Blasius	Cultivar	Saatzucht Donau, Austria
Bruehl	Cultivar	Washington State University, USA
Capo	Cultivar	Probstdorfer Saatzucht, Austria
Christoph	Cultivar	Saatzucht Donau, Austria
Dropia	Cultivar	Romania
DW-Red	Cultivar	University Idaho, USA
Ehogold	Cultivar	Saatzucht Edelhof, Austria
Estevan	Cultivar	Saatzucht Edelhof, Austria
Genius	Cultivar	Nordsaat, Germany
Globus	Cultivar	Nordsaat, Germany
Golden Spike	Cultivar	Utah State University, USA
Kolompos	Cultivar	Hungary
Lewjain	Cultivar	Washington State University, USA
Lukullus	Cultivar	Saatzucht Donau, Austria
Mirastar	Cultivar	Saatzucht Donau, Austria
Mulan	Cultivar	Nordsaat, Germany
MV Lucilla	Cultivar	Hungary
Nemchinovskaja 17	Cultivar	Russia
Pannonikus	Cultivar	Saatzucht Donau, Austria
Quebon	Cultivar	Nordsaat, Germany
Spontan	Cultivar	Secobra Saatzucht, Germany

Tillexus	Cultivar	Saatzucht Donau, Austria
Tillico	Cultivar	Getreidezüchtungsforschung Darzau, Germany
Tillstop	Cultivar	Saatzucht Donau, Austria
Tobias	Cultivar	Saatzucht Donau, Austria
Tommi	Cultivar	Nordsaat, Germany
Viki	Cultivar	InterSaatzucht GmbH, Germany
Weston	Cultivar	University Idaho, USA
**Blizzard**[1]	**Cultivar**	**University Idaho, USA**
**Bonneville**[1]	**Cultivar**	**University Idaho, USA**
**Rainer**[2]	**Cultivar**	**Saatzucht Donau, Austria**
**Midas**[2]	**Cultivar**	**Saatzucht Donau, Austria**
Akteur-M56	Experimental line	Saatzucht Donau, Austria
1314.3.11_P1	Experimental line	BOKU, Austria
1325.1.10_P1	Experimental line	BOKU, Austria
1351.5.10_P2	Experimental line	BOKU, Austria
**20568.1.2**[2]	**Experimental line**	**BOKU, Austria**
20812-2-2	Experimental line	BOKU, Austria
702-1102C	Experimental line	Nordic Seed, Denmark
P106.51.10	Experimental line	BOKU, Austria
W10.115.4.2.9	Experimental line	BOKU, Austria
W10.193.1.1.9	Experimental line	BOKU, Austria
W12.207.2.3.9	Experimental line	BOKU, Austria
W6.175.2.5.9	Experimental line	BOKU, Austria
PI 166910	Gene bank accession	USDA Genebank Aberdeen, Idaho, USA
PI 178383	Gene bank accession	USDA Genebank Aberdeen, Idaho, USA
PI 362695	Gene bank accession	USDA Genebank Aberdeen, Idaho, USA
PI 476212	Gene bank accession	USDA Genebank Aberdeen, Idaho, USA
PI 636156	Gene bank accession	USDA Genebank Aberdeen, Idaho, USA
PI 636165	Gene bank accession	USDA Genebank Aberdeen, Idaho, USA
PI 636170	Gene bank accession	USDA Genebank Aberdeen, Idaho, USA

[1] Resistance donor of mapping population

[2] Recipient parent of mapping or validation population

Table S3 Disease reaction of common bunt (CB) and dwarf bunt (DB) spore mixtures used for artificial inoculation. CB and DB incidence (%) of bunt differential lines *Bt1* to *Bt13* and *BtP*, parental lines and check cultivars

Experiment		CB composite			DB composite	
		CB.f15	CB.f16	CB.gh16	DB.f15	DB.f16
Bt differential line	*Bt* gene					
Selection 2092	*Bt1*	0.0	1.9	0.0	**16.0**	**21.4**
Selection 1102	*Bt2*	**60.2**	**44.5**	**83.1**	3.3	**23.7**
Ridit	*Bt3*	1.6	2.0	9.1	2.1	8.9
CI 1558B	*Bt4*	3.7	2.2	4.7	**31.7**	**45.3**
Hohenheimer	*Bt5*	0.0	0.0	0.0	0.5	2.6
Rio	*Bt6*	1.8	0.2	**12.2**	**15.1**	**59.5**
Selection 50077	*Bt7*	**71.5**	**51.5**	**62.2**	**10.3**	**39.2**
M82-2161	*Bt8*	1.1	0.2	**40.4**	0.0	0.5
M90-387	*Bt9*	0.0	0.0	**52.9**	0.8	7.3
M82-2102	*Bt10*	0.0	0.2	0.0	0.0	5.7
M82-2123	*Bt11*	0.0	0.5	0.0	0.0	0.1
PI 119333	*Bt12*	0.0	0.2	2.8	0.5	1.3
Thule III	*Bt13*	6.8	2.5	**35.0**	0.3	1.6
PI 173437	*BtP*	1.1	1.0	**15.5**	0.3	1.8
Capo	susceptible check	**62.1**	**81.2**	**100.0**	**37.9**	**60.4**
Midas	susceptible check	**80.1**	**79.4**	NA	**29.4**	**62.4**
Pannonikus	susceptible check	**69.3**	**68.7**	NA	**35.5**	**73.3**
Saturnus	susceptible check	**33.1**	**31.3**	NA	**10.3**	**32.1**
Globus	resistant check	0.0	3.4	NA	3.3	**26.0**
Goldens Spike	resistant check	0.0	0.0	NA	0.3	0.0
Weston	resistant check	0.0	0.0	NA	0.3	1.9
Blizzard	resistant parent	1.3	0.0	1.9	0.3	0.9
Bonneville	resistant parent	0.0	0.0	0.0	0.0	0.5
Rainer	susceptible parent	**81.2**	**82.0**	**92.2**	**18.5**	**38.8**

Table S4 Variance component estimates (σ^2) of genotype, environment, block, genotype by environment interaction and residual effects for common bunt (CB) and dwarf bunt (DB) incidence in population MP-BLI and MP-BON

	**Population MP-BLI**				**Population MP-BON**			
	CB incidence (%)		DB incidence (%)		CB incidence (%)		DB incidence (%)	
	Variance	SE	Variance	SE	Variance	SE	Variance	SE
σ^2 Genotype	535.01	73.46	98.52	15.41	541.74	85.96	108.37	18.86
σ^2 Block	0.00	0.00	1.48	1.70	2.07	2.05	5.63	5.95
σ^2 Environment	46.83	47.74	33.52	48.92	34.26	36.37	16.62	28.16
σ^2 Gen x Env	88.76	9.83	25.22	5.15	73.88	10.15	18.54	5.06
σ^2 Error	33.00	2.46	26.30	2.40	38.36	3.32	28.17	2.96

Table S5.1 Pearson's correlation coefficients between RIL bunt incidence BLUEs for all common bunt (CB) and dwarf bunt (DB) bunt environments analysed in mapping populations MP-BLI

		CB incidence (%)		DB incidence (%)	
		CB.f15	CB.f16	DB.f15	DB.f16
CB.gh16	r	0.80	0.83	0.63	0.49
	p.value	0.00	0.00	0.00	0.00
CB.f15	r		0.95	0.70	0.53
	p.value		0.00	0.00	0.00
CB.f16	r			0.68	0.50
	p.value			0.00	0.00
DB.f15	r				0.71
	p.value				0.00

Table S5.2 Pearson's correlation coefficients between RIL bunt incidence BLUEs across common bunt (CB) and dwarf bunt (DB) environments analysed in mapping population MP-BLI

		DB incidence (%)
CB incidence (%)	r	0.66
	p.value	0.00

Table S6.1 Pearson's correlation coefficients between RIL bunt incidence BLUEs for all common bunt (CB) and dwarf bunt (DB) environments analysed in mapping population MP-BON

		CB incidence (%)		DB incidence (%)	
		CB.f15	CB.f16	DB.f15	DB.f16
CB.gh16	r	0.84	0.84	0.24	0.37
	p.value	0.00	0.00	0.03	0.00
CB.f15	r		0.95	0.28	0.40
	p.value		0.00	0.01	0.00
CB.f16	r			0.28	0.40
	p.value			0.01	0.00
DB.f15	r				0.83
	p.value				0.00

Table S6.2 Pearson's correlation coefficients between RIL bunt incidence BLUEs across common bunt (CB) and dwarf bunt (DB) environments analysed in mapping population MP-BON

		DB incidence (%)
CB incidence (%)	r	0.38
	p.value	0.00
